# Neuromyelitis optica causing vision loss during TB treatment with sutezolid: evidence of aberrant immunity following infection

**DOI:** 10.5588/ijtldopen.25.0071

**Published:** 2025-05-12

**Authors:** N.A. Glover, L. Paramo, S. Giangregorio, B. Sukwana, S.E. Koele, S. Charalambous, T. Ngwanto, S. Mashatole, D. Mudzengi, N. Heinrich, E.M. Svensson, F. Abdullah, G. Churchyard, R.S. Wallis

**Affiliations:** ^1^ Aurum Institute, Johannesburg, South Africa;; ^2^ Institute of Infectious and Tropical Diseases, LMU University Hospital, German Center for Infection Research (DZIF), Munich Partner site, Munich, Germany;; ^3^ Department of Neurology, University of Pretoria, Pretoria, South Africa;; ^4^ Department of Pharmacy, Radboud Institute for Medical Innovation, Radboud University Medical Center, Nijmegen, the Netherlands;; ^5^ Vanderbilt University Medical Center, Nashville, TN, USA;; ^6^ Department of Pharmacy, Uppsala University, Uppsala, Sweden;; ^7^ South African Medical Research Council, Johannesburg, South Africa;; ^8^ Departments of Internal Medicine and Public Health Medicine, University of Pretoria, Pretoria, South Africa;; ^9^ School of Public Health, University of the Witwatersrand, Johannesburg, South Africa;; ^10^ Case Western Reserve University School of Medicine, Cleveland, OH, USA.

**Keywords:** tuberculosis, neuromyelitis optica, NMO, autoimmune, TB complications, oxazolidinone, drug toxicity

Dear Editor,

Adverse events during TB treatment are commonly attributed to drug toxicity or disease progression. The oxazolidinone antimicrobial drug, linezolid (LZD), is essential for the treatment of rifampicin-resistant TB, despite neurologic and ophthalmologic toxicities attributed to the inhibition of mitochondrial protein synthesis (MPS) in nerve cells.^1^ Newer oxazolidinones with reduced toxicity may permit the expanded use of these drugs in ‘pan-TB’ regimens.^2^ A new oxazolidinone, sutezolid (SZD; PNU-100480), is a thiomorpholinyl LZD analogue, with superior anti-TB activity in preclinical studies.^3^ Here, we report on a patient with pulmonary TB enrolled in a clinical trial of SZD who experienced Neuromyelitis optica (NMO), causing rapid vision loss and paralysis. NMO spectrum disorder is an uncommon antibody-mediated demyelinating disease manifesting as episodes of optic neuritis and transverse myelitis, often following viral or bacterial infections.^4^

The individual was 20–30-years-old with moderately advanced rifampicin-susceptible TB^5^ without prior neurologic disease, enrolled in the pan-TB-HM trial (NCT05686356). After providing written informed consent, the patient received SZD (1,600 mg daily), *N*-acetylcysteine (NAC; 1,800 mg twice daily) and bedaquiline and pretomanid at approved doses. Sputum obtained on Days 0, 1 and 7 confirmed growth of *Mycobacterium tuberculosis*; cultures were negative on Day 14 and subsequently. The individual’s weight increased from 42 to 50 kg until Week 7, when the onset of nausea, emesis, hiccups and blurred vision developed (Figure panel A). The patient initially sought advice from a traditional healer, although treatment was reportedly declined. Visual acuity was not initially measured. The serum anion gap reached 30 mEq/l, with relatively preserved bicarbonate levels. No aetiology was established for the illness. Anti-emetic treatment proved ineffective, and complete vision loss rapidly ensued 10 days after symptom onset. The participant was hospitalised, study treatments discontinued, and TB treatment changed to isoniazid plus rifampicin. On examination, the pupils were fixed and dilated, with normal fundi. Sensation and strength in the extremities were normal. Contrast-enhanced computed tomography of the head showed sphenoid pneumosinus dilatans but no other abnormalities. Magnetic resonance imaging (MRI) of the head showed diffuse symmetrically increased T2 signal throughout both optic nerves from the pre-chiasmal through retrobulbar portions, with corresponding diffuse gadolinium enhancement of the T1 signal of these same areas (Figure panel B). The optic nerve sheath diameter was 4.5 mm bilaterally in the mid portion (normal ∼5.0 mm). No other abnormalities were noted. The imaging diagnosis was bilateral optic neuritis. Lumbar cerebrospinal fluid showed 10 lymphocytes/µL but no other abnormalities. Oligoclonal band testing was not performed. Hyponatremia and high urine osmolarity were noted, consistent with the syndrome of inappropriate anti-diuretic hormone (SIADH). Multiple studies were normal or negative, including serum B_12_, syphilis serologies, PCR for HIV-1 and agents of viral meningitis, and IgM antibodies for Epstein-Barr virus, herpes simplex virus and rubella. Serum antibody tests for aquaporin 4 (AQP4) and myelin-oligodendrocyte glycoprotein (MOG) were negative. Treatment was initiated with methylprednisolone (1 g daily), water restriction and electrolyte support. Vision slowly improved to light perception and finger counting. Azathioprine was started. One day prior to discharge the participant described paresthesias on his torso. The discharge diagnosis was AQP4-negative NMO. New leg weakness was reported by the participant shortly thereafter, approximately 4 weeks after the last SZD dose. Weakness was initially one-sided but became bilateral, preventing arising from a chair or unassisted walking. Bowel or bladder incontinence was not reported. A second traditional healer evaluation was sought by the participant’s mother, who strongly discouraged hospital readmission. Azathioprine and TB treatments were reportedly continued. After multiple unsuccessful attempts to arrange further medical evaluation, contact ceased at the mother’s request.

**Figure.**
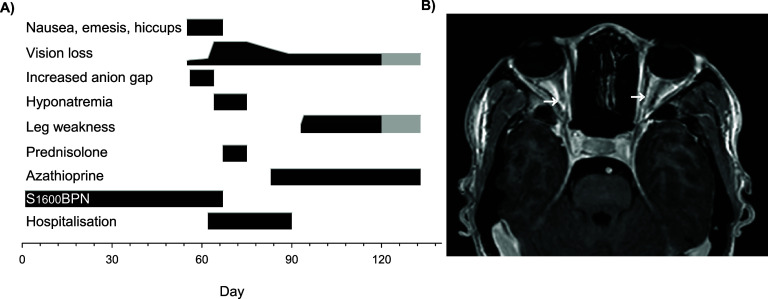
A: Case illness manifestations over time. S_1600_ = sutezolid 1600mg daily. B = bedaquiline. P = pretomanid. N = N-acetylcysteine. Bars indicate presence of illness, with amplitude corresponding to severity. Gray shading indicates lack of direct confirmation. B: Post-gadolinium T1-weighted MRI image showing enhancement of the optic nerves bilaterally (arrows).

Exploring the potential role of the investigational treatments, we focus on SZD and *N*-acetylcysteine. Optic neuropathy is an uncommon adverse effect of LZD treatment, most often occurring in patients with multidrug-resistant TB (MDR-TB).^1^ Risks increase in relation to cumulative LZD dose and trough plasma concentrations: in one small study, mitochondrial toxicity occurred in all patients with LZD trough levels >2 mg/L.^6^ Most reported cases of LZD optic neuropathy evolve over several weeks and improve after treatment is stopped. Sparse reports describe normal-appearing optic nerves on MRI, as is typical of toxic optic neuropathies generally.^7–9^ LZD more commonly causes peripheral neuropathy, with greater sensory than motor involvement. No cases have been reported to arise *de novo* weeks after drug discontinuation. Thus, key features of this case are unlike the neurological toxicities of LZD. Clinical experience to date suggests a reduced risk of adverse events with SZD due to MPS inhibition: no cases of anaemia, peripheral neuropathy, or optic neuropathy due to SZD have occurred in this clinical trial or in others to date.^10–12^ The low body weight of the participant may have resulted in high SZD exposures at the assigned dose (1,600 mg daily). A population pharmacokinetic model developed as a part of the PanACEA SUDOCU trial (NCT03959566) was used to simulate concentrations of SZD and its main metabolite (U-603) based on dose, body weight and adherence to treatment (Supplementary Table S1).^13^ They show relatively little impact of body weight on drug exposures within the investigated range. In none of the simulations did trough concentrations of SZD or U-603 exceed 2 mg/L, either individually or in combination. Although the corresponding threshold for SZD is not known, it is likely greater than that of LZD, based on its higher threshold for MPS inhibition. This participant also received NAC (1,800 mg twice daily). NAC is not known to cause optic neuropathy, even when administered chronically at substantially higher doses.^14^ NAC provides cysteine for synthesis of glutathione, a key antioxidant that otherwise becomes severely depleted in TB patients.^15^ SZD undergoes oxidation to an active sulfoxide metabolite (U-603) that circulates at levels several times those of the parent (Supplementary Table S1). The metabolite shows greater MPS inhibition in vitro compared to the parent (Pfizer, New York, NY, USA; unpublished), and in this respect, it is more like LZD than SZD. Thus, if NAC were to have any effect on these processes, it would tend to reduce levels of the U-603 metabolite, potentially reducing rather than increasing MPS risks. It therefore seems unlikely that NAC contributed to this event.

Neuromyelitis optica spectrum disorder (Devic’s disease) is an uncommon autoimmune disease of the eye and central nervous system.^16^ In 2004, antibody against AQP4, an astrocyte water channel, was identified as the main pathogenic mechanism.^17^ Approximately one-quarter of cases remain antibody-negative despite repeated testing, implicating other as-of-yet unidentified antigens or pathogenic mechanisms. In 2015, an expert panel described the core clinical characteristics of NMO:^18^

• Optic neuritis, with MRI showing T2-hyperintense lesion or T1-weighted gadolinium-enhancing lesion over >1/2 optic nerve length or optic chiasm involvement, with otherwise normal or non-specific white matter findings
• Longitudinally extensive transverse myelitis, with MRI showing involvement of ≥3 contiguous vertebral segments, with both sensory and motor findings on exam
• Area postrema syndrome (APS), with intractable nausea, emesis and hiccups, due to involvement of dorsal medulla
• Brainstem syndrome (APS plus nystagmus or diplopia) due to brainstem involvement
• Narcolepsy (sudden involuntary episodes of sleep).

In the presence of AQP4 antibody, any single core clinical characteristic is sufficient to establish the diagnosis; in its absence, at least two core characteristics must be present, with characteristic lesions identified by MRI in at least two areas of the central nervous system (CNS).^18^

A growing list of viral and bacterial infections have been reported as triggers of NMO episodes, including multiple reports of TB-associated cases.^3,19,20^ A case-control study in South Africa found an association between NMO and recent pulmonary TB (odds ratio 4.6, 95% confidence interval 1.7–15.5);^21^ a similar study in China did not, possibly due to its substantially lower TB incidence.^22^ Mimicry between mycobacterial and host antigens has been implicated as the underlying mechanism. This case also included SIADH and increased anion gap. SIADH is a common manifestation of many illnesses involving the CNS. Although SIADH is not among the diagnostic criteria for NMO, multiple reports describe its presence.^23,24^ Thus, the features of this case are highly consistent with NMO, and inconsistent with oxazolidinone neurologic toxicity. The clinical course of NMO is typically one of distinct episodes involving different regions of the CNS with some degree of improvement between episodes, but neither progression nor full recovery are common. The prognosis without treatment is poor: by 5 years, half of patients will be blind and wheel-chair bound, and a third will have died.^16^ Aggressive treatment with plasma exchange in a brief time window is recommended early during attacks. Immunosuppressive therapy with anti-B cell agents is recommended to prevent recurrences.^25^

In summary, neurologic symptoms due to aberrant immunity may arise during otherwise successful TB treatment. There is increasing recognition that NMO, a demyelinating disease affecting the optic nerves and spinal cord, may be triggered by bacterial infections, including TB. In such cases, prompt recognition and aggressive immunosuppressive therapy may help prevent lifelong disability.

## Supplementary Material


