# Supplementary material for: Neuromyelitis optica causing vision loss during TB treatment with sutezolid: evidence of aberrant immunity following infection
Source: IJTLD Open. 2025 May 12;2(5):306–9. doi: 10.5588/ijtldopen.25.0071 (PMC12068453; doi:10.5588/ijtldopen.25.0071)
Supplement: Supplementary file 1 [file ijtldopen25-0071_supplementarydata1.pdf]

**Supplemental materials**

Table S1. PK modelling of sutezolid and U-603 according to body weight.

| | AUC0-24 [mg/L*h] | | Cmax [mg/L] | | Cmin [mg/L] | |
|---|---|---|---|---|---|---|
| Scenario | Sutezolid | U-603 | Sutezolid | U-603 | Sutezolid | U-603 |
| 40KG | 12.2 (5.85-25.4) | 63.9 (40.6-106) | 1.91 (0.83-4.27) | 7.57 (4.15-14.1) | 0.13 (0.06-0.28) | 0.86 (0.51-1.38) |
| 50KG | 10.3 (4.95-21.5) | 54.1 (34.3-89.4) | 1.57 (0.68-3.49) | 6.19 (3.42-11.5) | 0.11 (0.05-0.24) | 0.73 (0.44-1.18) |
| Patient at NMO onset (50 kg) | 9.99 (4.71-21.4) | 52.5 (33.1-87.7) | 1.54 (0.66-3.48) | 6.09 (3.32-11.4) | 0.1 (0.05-0.22) | 0.68 (0.41-1.09) |

Abbreviations: AUC0-24, area under the concentration curve from time 0 to 24 after dose; Cmax, maximal concentration in the dosing interval; Cmin, minimal concentration in the dosing interval, e.g. trough concentration; NMO, neuromyelitis optica; PK, pharmakokinetic. Values indicate median and 95% confidence intervals representing expected inter-individual variability. 10,000 virtual patients were simulated for each scenario.
